# A Locus on Chromosome 5 Is Associated with Dilated Cardiomyopathy in Doberman Pinschers

**DOI:** 10.1371/journal.pone.0020042

**Published:** 2011-05-20

**Authors:** Theresa-Bernadette Mausberg, Gerhard Wess, Julia Simak, Lisa Keller, Michaela Drögemüller, Cord Drögemüller, Matthew T. Webster, Hannah Stephenson, Joanna Dukes-McEwan, Tosso Leeb

**Affiliations:** 1 Institute of Genetics, Vetsuisse Faculty, University of Bern, Bern, Switzerland; 2 Clinic of Small Animal Medicine, LMU University, Munich, Germany; 3 Department of Medical Biochemistry and Microbiology, Uppsala University, Uppsala, Sweden; 4 Small Animal Teaching Hospital, University of Liverpool, Liverpool, United Kingdom; Istituto Dermopatico dell'Immacolata, Italy

## Abstract

Dilated cardiomyopathy (DCM) is a heterogeneous group of heart diseases with a strong genetic background. Currently, many human DCM cases exist where no causative mutation can be identified. DCM also occurs with high prevalence in several large dog breeds. In the Doberman Pinscher a specific DCM form characterized by arrhythmias and/or echocardiographic changes has been intensively studied by veterinary cardiologists. We performed a genome-wide association study in Doberman Pinschers. Using 71 cases and 70 controls collected in Germany we identified a genome-wide significant association to DCM on chromosome 5. We validated the association in an independent cohort collected in the United Kingdom. There is no known DCM candidate gene under the association signal. Therefore, DCM in Doberman Pinschers offers the chance of identifying a novel DCM gene that might also be relevant for human health.

## Introduction

Cardiomyopathies are a heterogeneous group of diseases, which can be inherited or associated with specific cardiac or systemic disorders. Human primary cardiomyopathies are usually divided into five main pathological groups: dilated, hypertrophic, restrictive, arrhythmogenic right ventricular, and unclassified cardiomyopathies [1]. They may be caused by one or several genetic mutations. Alternatively cardiomyopathies may be idiopathic, or secondary post-inflammatory, following a generalized systemic disorder. In humans the prevalence of dilated cardiomyopathy (DCM) is 36.5/100,000 [2]. Inherited forms make up to 35% of the cases [3] and are mostly inherited as monogenic autosomal dominant traits [4].

Dogs are excellent genetic models for human diseases as they share many hereditary diseases with humans. Purebred dog breeds represent isolated populations with extreme genetic bottlenecks at breed foundation ∼200 years ago. Therefore the heterogeneity within a breed is greatly reduced, which facilitates the identification of causative mutations for complex diseases [5], [6]. Another advantage of dogs as a model for DCM is that they can be examined with sophisticated diagnostic equipment in a very similar way to humans, so that the pathophysiological alterations of the heart are relatively well understood.

Large dogs are frequently affected by cardiomyopathies and in the Doberman Pinscher a specific form of DCM is known. The Doberman DCM is inherited as an autosomal dominant trait similar to most human DCM forms [7]. The prevalence of Doberman DCM in Europe is 58.2% [8]. Electrical aberrations like ventricular arrhythmias are typical for Doberman DCM and occur together with or even before morphological changes of the heart. Frequently, affected Doberman Pinschers suffer a sudden death due to these arrhythmias and do not reach the stage where they develop a congestive heart failure [9]–[12]. Age of onset can be as early as one year, but eight year old dogs can also still develop DCM. The average age at which DCM affected Dobermans develop congestive heart failure is 6.7 years [9]. The progression of Doberman DCM can be divided into 3 stages. In stage I, the morphology of the heart is normal and there are no clinical signs of heart disease or electrical derangements. However, a genetic defect is assumed to exist, which gradually causes damage on the cellular level. This stage can last for months or years. [13]. In stage II, morphological and electrical changes occur, but there are still no clinical signs of the heart disease. From the owner's point of view, there are no signs of DCM and the dog seems to be healthy. Therefore, this stage is called “the occult stage”. During Stage II morphological and electrical abnormalities may coexist, or one form may be predominant at any time. About 30% of the affected dogs die due to sudden death in this stage, which typically lasts 3 to 4 years. Finally, affected dogs enter stage III, which is also called the “overt stage” of the Doberman DCM. Often, this is the first time that the owners realize that their dogs may have heart problems. [13], [14]. In stage III, affected dogs develop congestive heart failure and the typical clinical symptoms like tachycardia, tachypnea, and cough. Stage III may last days to years until the dogs die due to sudden death or as a result of the congestive heart failure [13], [14].

In humans, mutations in at least different 19 genes may cause hereditary forms of DCM [15], [16]. Many of these genes encode structural proteins of the heart muscle. However, there are still many human DCM forms, where the causative mutation remains unknown. Using a comparative approach, many of the known human genes and some functionally related genes were evaluated in Dobermans. So far 15 genes (*ACTC1, CAV1, CSRP3, DES, LDB3, LMNA, MYH7, PLN, SGCD, TCAP, TNNC1, TNNI3, TNNT2, TPM1, VCL*) were studied, but did not reveal the causative mutation for Doberman DCM [17]–[22]. Therefore, the identification of the causative mutation of the Doberman DCM might identify a novel DCM gene that is also relevant for human DCM forms, where the causative mutations have not been identified so far. In this study we used a hypothesis-free approach and performed a genome-wide association study (GWAS) to map genetic risk factors for DCM in Doberman Pinschers.

## Results

### Phenotypical characterization

We sampled Doberman Pinschers in Germany and the UK. We compiled a discovery cohort consisting of 71 DCM affected and 70 DCM control dogs from Germany. All dogs in the discovery cohort were examined by Holter electrocardiogram (24 hour ECG) and echocardiogram. DCM in Doberman Pinschers can present with electrical derangements, such as arrhythmias that can be monitored by the Holter electrocardiogram and/or with morphological changes, such as a dilation of the heart that can be seen by echocardiogram. Many Doberman Pinschers with DCM show a combination of both symptoms. Our discovery cohort consisted of 19 cases with isolated arrhythmias, 9 dogs with isolated echocardiographic abnormalities, and 43 dogs with a combination of arrhythmias and echocardiographic abnormalities. The control group of the discovery cohort consisted of Doberman Pinschers older than 6 years of age with normal Holter electrocardiogram and normal echocardiographic findings. We further collected a validation cohort consisting of 15 Doberman DCM cases and 24 Doberman controls from the United Kingdom. In the validation cohort one case had isolated arrhythmias and 14 cases had a combination of arrhythmia and echocardiographic changes.

### Mapping of the genome-wide association

We genotyped 174′376 SNP markers in the discovery cohort consisting of 71 DCM cases and 70 controls. After removing non-informative markers and markers with low call rates, 75′916 markers remained for the final analysis. The genomic inflation factor was 1.07 indicating that the samples were only minimally stratified. We initially performed a genome-wide allelic association study with the DCM phenotype. We detected a genome-wide significant association on canine chromosome 5 (CFA 5). The best-associated SNP, TIGRP2P73097 (CFA5:g.53,941,386T>C), showed a raw p-value of 8.36×10^−8^ and a genome-wide corrected p-value of 0.0071. There were no significant associations on any of the other chromosomes.

We then analyzed the association to either the echocardiographic changes or the arrhythmias separately. For the echocardiographic changes we removed the 19 DCM cases with isolated arrhythmias and compared the remaining 52 cases to the 70 controls. CFA5:g.53,941,386T>C remained the best-associated SNP, but the genome-wide corrected p-value increased to 0.0503.

**Figure 1:**
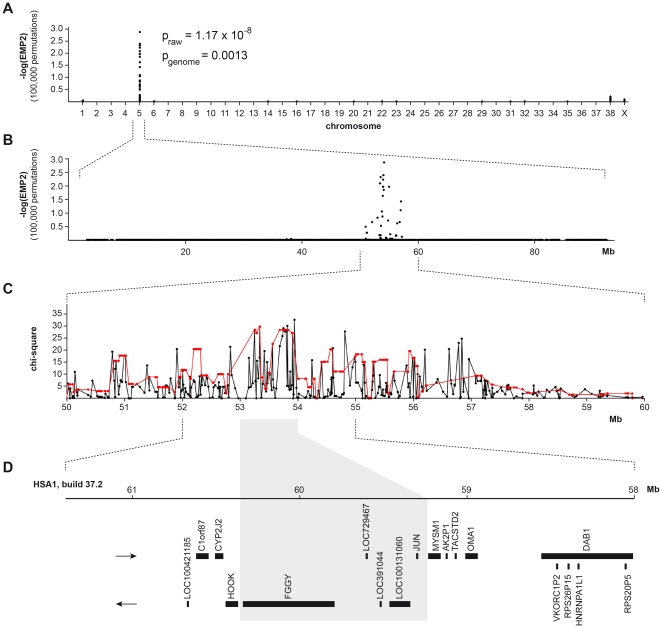
Genome-wide association mapping of Doberman DCM. (**A**) A case-control genome-wide allelic association analysis in the discovery cohort showed a significant association of the phenotype arrhythmia in 62 cases and 70 controls to SNPs on chromosome 5. (**B**) Several SNPs in a ∼7 Mb interval on chromosome 5 were associated. (**C**) The association of single SNPs (black) and haplotypes (red) at the DCM locus indicated that the most likely position for the DCM risk factor is between 53 Mb and 54 Mb. Haplotypes were estimated with the Haploview software and the “confidence intervals” option. (**D**) As the dog genome annotation is still fragmentary, we inferred the gene content by extrapolating from the human orthologous interval. The grey shaded area corresponds to the interval between 53 and 54 Mb on CFA 5.

In the GWAS with respect to the arrhythmias we used 62 cases and 70 controls. Using these cohorts the genomic inflation factor slightly increased to 1.13. For the phenotype arrhythmia we observed the most significant associations of all performed analyses (Figure 1). Twelve SNPs within a 3.6 Mb interval on CFA 5 had genome-wide corrected p-values of less than 0.05. The best-associated SNP was again CFA5:g.53,941,386T>C. It had an asymptotic raw p-value of 1.17×10^−8^ and a genome-wide corrected p-value of 0.0013. The odds ratio at this SNP was 8.15 with a 95% confidence interval ranging from 3.64–18.24.

The frequency of the DCM-associated C-allele at CFA5:g.53,941,386T>C varies among the different subphenotypes (Table 1). It is highest among the group of Dobermans with isolated arrhythmias and lower in Dobermans that have only echocardiographic changes. However, these differences in allele frequencies between the different DCM sub-phenotypes are not statistically significant (Fisher's exact test).

**Table 1:** Frequency of the DCM-associated C-allele at CFA5:g.53,941,386T>C in different subsets of the discovery cohort.

Subset	Dogs	Allele Frequency	[%]
Arrhythmia only	19	13/38	34.2%
Arrhythmia & echocardiographic changes	43	28/86	32.6%
Echocardiographic changes only	9	2/18	11.1%
Control dogs	70	8/140	5.7%

### Validation of the association

We genotyped the 10 best-associated SNPs from the arrhythmia GWAS also in the validation cohort (Table 2). In the validation cohort 9 of these 10 markers had p-values of less than 0.05. When analyzing the discovery cohort and validation cohort together the best combined raw p-value was 7.04×10^−10^ for CFA5:g.53,941,386T>C. The C-allele at this SNP is found more frequently in DCM cases in the discovery and the validation cohort.

**Table 2:** Validation of the 10 best-associated SNPs from the arrhythmia discovery cohort.

			Genome-wide analysis		Replication analysis		
			Allele frequencies (cases/controls)		Allele frequencies (cases/controls)		
Position	SNP	Alleles	Germany (62/70)	p_raw_ a	UK (15/24)	p_raw_ a	Combined p-value
53,941,386	TIGRP2P73097	C/T	0.33/0.06	1.17×10^−8^	0.13/0.02	0.048	7.04×10^−10^
53,820,695	TIGRP2P73026	T/C	0.31/0.05	4.27×10^−8^	0.11/0.02	0.104	3.87×10^−9^
53,344,207	BICF2P586593	C/G	0.31/0.06	4.87×10^−8^	0.13/0.02	0.048	3.06×10^−9^
53,751,794	BICF2P1162831	A/C	0.31/0.06	6.76×10^−8^	0.13/0.02	0.048	4.49×10^−9^
53,252,611	BICF2S2334544	T/C	0.31/0.06	9.84×10^−8^	0.13/0.02	0.048	6.32×10^−9^
53,252,611	TIGRP2P72998	A/G	0.31/0.06	1.43×10^−7^	0.13/0.02	0.048	9.09×10^−9^
53,776,936	TIGRP2P73008	G/C	0.31/0.06	1.43×10^−7^	0.13/0.02	0.048	9.09×10^−9^
53,796,343	TIGRP2P73009	G/A	0.31/0.06	1.43×10^−7^	0.13/0.02	0.048	9.09×10^−9^
53,797,457	BICF2S23229423	A/G	0.31/0.06	1.43×10^−7^	0.20/0.02	0.007	2.15×10^−9^
54,816,226	BICF2P1307722	A/G	0.30/0.06	1.97×10^−7^	0.13/0.02	0.048	1.30×10^−8^

a p-values were calculated by χ^2^ tests in an allelic association study.

**Figure 2:**
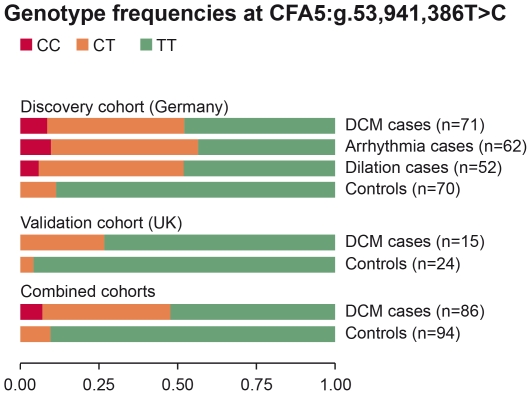
Genotype frequencies at the best associated SNP TIGRP2P73097. The genotype distributions indicate a dominant or additive effect of the disease-associated variant.

The genotype frequencies at this SNP are displayed in figure 2. Among the combined 86 DCM cases of both cohorts 41 Dobermans (48%) were either homozygous or heterozygous for the risk-allele. However, the other 45 DCM cases (52%) were homozygous for the protective allele. Among the combined 94 Doberman controls, only 9 dogs (10%) carried the risk-allele in heterozygous state, and none of the controls was homozygous for the risk-allele.

### Positional candidate gene analysis

As the annotation of the canine genome is still far from perfect, we inferred the gene content of a 3 Mb interval around the association signal from the corresponding human interval (Figure 1D). This interval corresponds to a segment from 58.0–61.4 Mb on HAS 1. The human interval contains 14 annotated genes and 4 hypothetical loci (NCBI MapViewer, build 37.2). A careful inspection of these genes and database searches of their presumed function revealed that none of them represents a known DCM gene. The “FGGY carbohydrate kinase domain containing” gene (*FGGY*) in the associated interval is a protein coding gene with unknown function. We sequenced its 15 coding exons in four affected and four control Doberman Pinschers and compared the sequences to the Boxer reference genome [23]. We identified 14 polymorphisms in total (Table S1). One variant was a silent polymorphism in exon 10, the other 13 polymorphisms were located in introns. At the exonic polymorphism all eight Doberman Pinschers were homozygous for the variant allele, whereas the Boxer sequence represented the presumed wildtype allele. We typed four intronic polymorphisms in the discovery cohort, but their association was at least four orders of magnitudes weaker than CFA5:g.53,941,386T>C.

## Discussion

We detected a significant genome-wide association of several SNPs on CFA 5 to DCM in Doberman Pinschers from Germany. The association could be confirmed in an independent cohort of Doberman Pinschers from the UK. Our findings confirm the value of purebred dogs to elucidate genetic risk factors for complex diseases. In humans DCM is extremely heterogeneous and very large cohorts are required to detect associations to low frequency risk alleles, while extremely rare risk alleles may not be detectable at all in human genome-wide association studies [24]. Doberman Pinschers represent a closed population that was founded about 150 years ago from a limited number of individuals by the German Friedrich Louis Dobermann [25]. Due to their population history the heterogeneity of DCM is much smaller in Doberman Pinschers than in humans, which facilitates the genetic mapping of DCM risk loci. However, even within the Doberman Pinscher breed, DCM is not a simple monogenic trait where a single mutation with full penetrance is responsible for all cases. Our findings that approximately half of the DCM affected Doberman Pinschers carry the risk-allele on CFA 5 indicate that this is a major, but not the only genetic risk factor for DCM in this breed. The lack of other association signals further suggests that the unexplained 50% DCM cases might be caused by several different genetic risk factors of small effect size, which cannot be detected with the currently available cohort sizes.

The recent genetic bottleneck in the Doberman Pinscher breed is responsible for a relatively long-reaching linkage disequilibrium (LD) in this breed. The long LD facilitates the detection of causative mutations with a limited amount of markers. Unfortunately, it simultaneously limits the mapping resolution. Thus the true causative mutation underlying the detected association signal might be located anywhere in a 1 Mb region, which poses significant challenges for mutation identification, especially if the causative variant should be a non-coding mutation. We already excluded coding mutations of the *FGGY* gene.

Doberman DCM is characterized by two distinct clinical features. On the one hand arrhythmias are very typical in Doberman DCM and not seen so frequently in DCM forms of other large dog breeds. On the other hand some Doberman Pinschers with DCM present only with echocardiographic changes, but no arrhythmia. The latter group more closely resembles the clinical symptoms of DCM in other large dog breeds such as the Irish Wolfhound, Great Dane, or Newfoundland. The strong association to arrhythmia in our cohorts suggests that the risk factor on CFA 5 is primarily related to the arrhythmias. On the other hand, the weaker association of CFA 5 to echocardiographic changes might indicate that the heart dilation is indeed controlled by at least partially distinct genetic risk factors. The genetic dissection of the two sub-phenotypes is hampered by the fact that the majority of DCM affected Doberman Pinschers have a combination of both symptoms of varying severity and with a variable age of onset.

In conclusion, we have mapped a major genetic risk factor for DCM in the Doberman Pinscher to CFA 5. In the region of the association signal, no obvious DCM candidate genes are located. Thus the further investigation of this association signal has a high chance of identifying a new DCM gene that might also be of relevance for a fraction of the unexplained human DCM cases.

## Materials and Methods

### Ethics statement

The dogs in this study were examined during preventive diagnostic procedures with the consent of their owners. All local regulations (Germany and UK) were strictly observed. The study was approved by the University of Munich and the University of Liverpool Committees on Research Ethics, respectively. There is no permit number as this study is not based on an invasive animal experiment. The data were obtained during diagnostic procedures that would have been carried out anyway. This is a very special situation in veterinary medicine. As the data are from client-owned dogs that underwent normal veterinary exams, there was no “animal experiment” according to the legal definitions in Germany and the UK. Nonetheless, the study was reviewed and approved by the local ethics review boards.

### Animals

We sampled 71 DCM affected Doberman Pinschers (40 males and 31 females, mean age 7.9 years) and 70 unaffected Doberman Pinschers (28 males and 42 females, mean age 9.3 years) from Germany for the discovery cohort. Furthermore, we sampled 15 DCM affected Doberman Pinschers (10 males and 5 females, mean age 7.2 years) and 24 control Doberman Pinschers (7 males and 17 females, mean age 8.5 years) from the United Kingdom for the validation cohort.

### Cardiologic examinations

All dogs were examined without sedation in right and left lateral recumbency, scanning through the dependent thoracic wall. Echocardiographic examinations in Germany were performed using a commercially available high frame rate ultrasound system equipped with a 2.0/4.3 MHz probe (Vivid 7 dimension, GE Medical Systems) with simultaneous ECG recording by the ultrasound machine. Various echocardiographic machines with simultaneous ECG were used in the UK. M-mode measurements were obtained from the right parasternal long-axis view. All valves were examined using color-Doppler. Velocities over the aortic and pulmonary valves were measured using continuous wave Doppler examinations and had to be below 2.2 m/sec. For the German dogs 24 hour Holter recordings were performed at each exam and analyzed using one of two commercially available software programs (Custo tera, Arcon Systems GmbH; Amedtech ECGpro Holter software, EP 810 digital Recorder, Medizintechnik Aue GmbH). Manual adjustments and accuracy verification of the arrhythmias recognized by the software were performed by members (veterinarians) of the LMU cardiology team with extensive experience in Holter analysis and overseen by a Diplomate in cardiology (GW). Holter recordings were not mandatory in the UK, but when used, the Del Mar Reynolds Lifecard CF system (Del Mar Reynolds Medical Ltd. UK) was used with analysis performed by one of two commercial companies (LifeCorp USA or Holter Monitoring Services, UK).

### Exclusion and inclusion criteria

We grouped dogs of the discovery cohort into the case and control groups according to the results of the Holter and echocardiographic M-mode examinations at the last available examination:

#### Control group

Dogs in this group had to be older than 6 years of age, no clinical signs, <50 ventricular premature contractions (VPCs)/24 hours, and normal echocardiographic measurements. M-mode values that were considered to be normal were as follows: left ventricular internal end-diastolic dimension (LVIDd)≤47 mm and left ventricular internal end-systolic dimension (LVIDs)≤38 mm [14], [26].

#### DCM case group

DCM was diagnosed if either >100 VPCs/24 hours were detected on Holter examination or if echocardiographic M-mode examinations were indicative for DCM, or both (Holter and echocardiography) were abnormal. M-mode values that were considered abnormal and indicative of DCM were as follows: LVIDd≥49 mm [27], [28], or LVIDs≥40 mm [13], [14], or both measurements increased.

#### Arrhythmia case group

Dogs with >100 VPCs/24 hours.

#### Dilation case group

Dogs with: LVIDd≥49 mm or LVIDs≥40 mm, or both measurements increased.

Dogs with evidence of systemic disease, concomitant congenital heart disease or evidence of primary mitral valve disease (based on echocardiography) were not included. Dogs that had between 50 and 100 VPCs/24 hours were considered “equivocal” and were also not included in the study.

Dogs in the validation control cohort were≥7 years old, had normal echocardiogram and no arrhythmias identified during three minute electrocardiographic recording or during the echocardiographic study but were not required to have a Holter examination. The validation case cohort all had echocardiographic examinations, and 4/15 also had Holters with an additional 3 cases showing frequent ventricular premature complexes during echocardiography and electrocardiography.

### SNP genotyping

Genomic DNA was isolated from EDTA blood with the Nucleon Bacc2 kit (GE Healthcare). The DNA was genotyped at the Centre National de Génotypage, Evry, France using illumina canine_HD chips containing 174′376 SNP markers. Genotypes were stored in a BC/SNPmax Database version 3.4 (BC/Platforms). Targeted genotypes were determined by Sanger sequencing on an ABI 3730 capillary sequencer.

### Genome sequences

Genome sequences were downloaded using the NCBI Map Viewer genome browser [29]. The CanFam2.1 version of the dog genome and build 37.2 of the human genome reference assembly were used.

### Mapping of the association

We used the plink software for the genome-wide association analysis [30]. A total of 174,376 markers were initially considered for the analysis. No individual was removed for low genotyping success (–mind 0.1). After removing 1,911 SNPs which failed missingness test (–geno 0.1) the average genotyping rate per individual was 99.1%. A total of 97,382 SNPs had a minor allele frequency of less than 10% and were removed (–maf 0.1). An additional 778 markers that deviated from a relaxed Hardy-Weinberg equilibrium based on HWE test (p<1×10^−05^) were also excluded from further analysis. After frequency and genotyping pruning, 75,916 SNPs remained in the analysis.

A case-control analysis using the option –assoc was applied. Genome-wide corrected empirical p-values were determined applying the max(T) permutation procedure implemented in plink with 100,000 permutations (–mperm 100000). The genomic inflation factor based on the median chi-squared was obtained by applying the –adjust option.

During the fine-mapping single SNP associations and haplotype associations were calculated with Haploview [31]. Haplotype blocks were estimated using the “confidence intervals” option of Haploview.

### DNA sequencing and mutation analysis

The exon/intron structure of the canine *FGGY* gene was inferred by alignment of human mRNA sequence to the canine genomic sequence. For mutation analysis, PCR products were amplified from four DCM affected and four healthy control Doberman Pinschers using AmpliTaq Gold 360 Master Mix (Applied Biosystems). The PCR products were directly sequenced after rAPid alkaline phosphatase (Roche) and exonuclease I (New England Biolabs) treatment using both PCR primers with the ABI BigDye Terminator Sequencing Kit 3.1 (Applied Biosystems) on an ABI 3730 capillary sequencer. Sequence data were analyzed with Sequencher 4.9 (GeneCodes).

## Supporting Information

Table S1 Polymorphisms and genotypes of 4 DCM affected and 4 control Doberman Pinschers in the *FGGY* gene.
(XLS)
